# Predictive Performance of MRI for Antibiotic Treatment Failure of Pyogenic Vertebral Osteomyelitis: A Validation Study

**DOI:** 10.7759/cureus.47933

**Published:** 2023-10-29

**Authors:** Sugihiro Hamaguchi, Sei Takahashi, Yuji Endo, Yohei Nakamoto, Tetsuro Aita, Toru Naganuma, Hiroaki Nakagawa, Toshihiko Takada

**Affiliations:** 1 Department of General Internal Medicine, Fukushima Medical University, Fukushima, JPN; 2 Center for Innovative Research for Communities and Clinical Excellence (CiRC2LE), Fukushima Medical University, Fukushima, JPN; 3 Futaba Emergency and General Medicine Support Center, Fukushima Medical University, Fukushima, JPN; 4 Department of Orthopedic Surgery, Fukushima Medical University, Fukushima, JPN; 5 Department of General Medicine, Shirakawa Satellite for Teaching and Research (STAR), Fukushima Medical University, Shirakawa, JPN

**Keywords:** bone destruction, predictive performance, antibiotic treatment failure, magnetic resonance imaging, pyogenic vertebral osteomyelitis

## Abstract

Introduction: Intravenous antibiotics are the primary treatment of choice for pyogenic vertebral osteomyelitis (PVO). Surgical intervention is required when the initial antibiotic treatment fails but is often difficult to perform, especially in older adults with multiple comorbidities, because of the reduced physical activity. The size of the infection signal in the spinal bone on magnetic resonance imaging (MRI) at the time of diagnosis was reported to have a high predictive accuracy for antibiotic treatment failure. However, the sample size was too small for this result to be adopted in clinical practice. Thus, we conducted a validation study of the previous research using a larger sample size.

Methods: We conducted a retrospective review of electronic medical records of patients admitted to the orthopedic department of a university hospital with a diagnosis of PVO between 2006 and 2021, and consecutively included patients without planned PVO surgery on admission and with a sagittal view of T1-weighted spinal MRI at the time of diagnosis. The index test was the percentage involvement of the affected areas in one motion segment on sagittal MRI. We also evaluated other MRI findings, such as bone destruction, segmental instability, epidural abscesses, and multiple sites for their predictive accuracy for antibiotic treatment failure.

Results: A total of 82 participants were eligible for the analysis. The presence of ≥90% affected area of one motion segment had a sensitivity of 16.7% and a specificity of 70.3% for future antibiotic treatment failure, resulting in poor predictive performance, with positive (LR+) and negative likelihood ratios of 0.56 and 1.19, respectively. The area under the receiver operating characteristic curve for a 10% increase in the affected area was 0.48. Among the other MRI findings, the presence of bone destruction had a significantly higher predictive accuracy (LR+ 3.11, 95% confidence interval 1.30-7.42).

Conclusion: An infection signal ≥90% on a T1-weighted MRI of one spinal motion segment did not show sufficient predictive performance for antibiotic treatment failure. Spinal bone destruction had a mild-to-moderate predictive accuracy.

## Introduction

Pyogenic vertebral osteomyelitis (PVO) is an uncommon infectious disease occurring worldwide with a mean annual incidence of 4.8-7.8 cases per 100,000 individuals reported in the United States, Japan, and France [1-3]. In Japan, among those aged 80 years or older, the incidence rate of PVO is approximately 25.1 cases per 100,000 individuals [2]. This upward trend can be attributed to not only the susceptible aging population but also the increasing opportunities for bacteremia, including the use of intravascular devices, renal replacement therapy, and immunosuppressive medications [4,5]. With the aging of the world’s population, the prevalence of this condition is expected to continue rising.

Magnetic resonance imaging (MRI) of the spine is the most sensitive radiographic modality for the diagnosis of PVO [6], and a minimum of six weeks of targeted antibiotic therapy is the primary treatment [7]. However, timely surgical intervention is necessary for patients with suboptimal response to antibiotics, given the risk of neurological abnormalities and bacteremia due to epidural or paravertebral abscesses [5]. In older adults, particularly those with multiple comorbidities, when antimicrobial therapy fails, perioperative physical activity levels often decline, making surgery more challenging [8]. Even if surgery is successfully performed, it often results in postoperative complications and long-term physical dysfunction [9]. Early surgical intervention should be planned by predicting antibiotic treatment failure at the time of diagnosis.

Although research on the prediction of antibiotic treatment failure at the time of diagnosis using MRI is scarce, Hodges et al. showed that MRI can predict the need for early surgical intervention [10]. In their study, an area ≥90% and an infection signal on T1-weighted MRI of one spinal motion segment comprising one intervertebral disc and two vertebral bodies had a sensitivity and specificity of 78% and 93%, respectively, in predicting future antibiotic treatment failure. However, the study was based on a limited cohort of 22 patients. Consequently, the predictive accuracy for prognosis requires further validation using a larger dataset before application in clinical practice. To validate the predictive accuracy of the affected area of one spinal motion segment on spinal MRI for antibiotic treatment failure, we conducted a retrospective analysis using a dataset of patients hospitalized with PVO at a single tertiary hospital.

## Materials and methods

This retrospective observational study was conducted at a single referral hospital and was approved by the Research Ethics Committee of Fukushima Medical University School of Medicine (approval number: REC2022-023). The ethics committee waived the need for written informed consent because this study was retrospective. We followed the Standards for Reporting of Diagnostic Accuracy 2015 (STARD 2015) statement for diagnostic accuracy studies [11].

Setting and patients

This study was conducted at a university hospital in Fukushima Prefecture, Japan, with a bed capacity of 778. It is the largest referral center for orthopedic surgeries, including spinal procedures, in the prefecture. Patients with PVO with the indication of treatment were referred to the center. We retrospectively reviewed the electronic medical records of patients admitted to the orthopedic department with a diagnosis of PVO between 2006 and 2021. We consecutively included patients aged 18 years or older who had no planned PVO surgeries on admission and who had undergone an MRI of the spinal bones, including sagittal view plain T1-weighted imaging at the time of diagnosis. Patients who underwent surgical intervention on the day of hospital admission were excluded even if there was no plan for surgery at admission. In addition, those with spinal device infection and postoperative infection were further excluded for having indeterminable MRI that hindered accurate assessment of osteomyelitis. Demographic and clinical data were also obtained, including age, sex, underlying comorbidities (diabetes mellitus, cirrhosis, end-stage renal diseases, hypertension, cardiovascular diseases, cerebrovascular diseases, rheumatologic disorders, and malignancy), and C-reactive protein.

Index test

**Figure 1:**
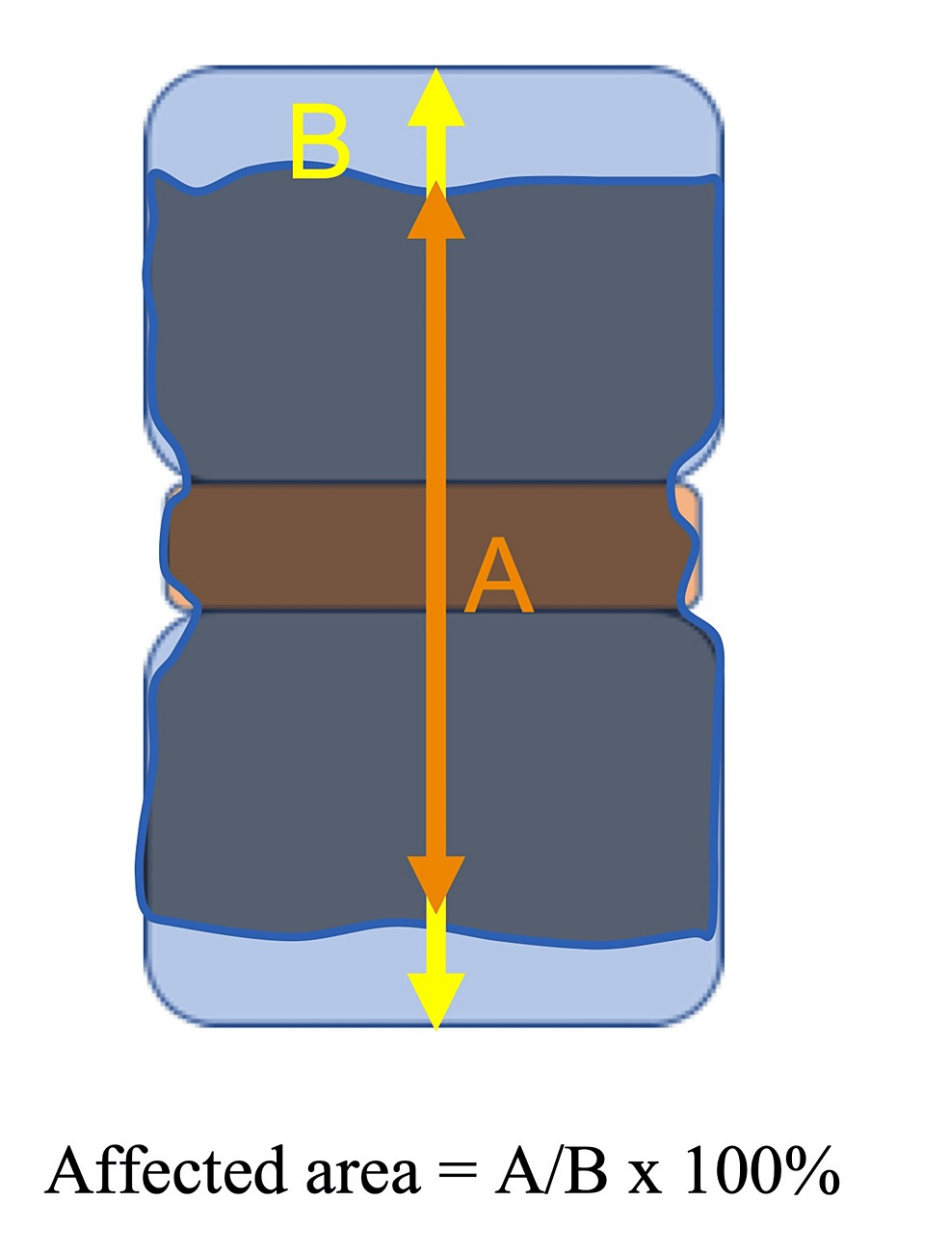
The measurement method of an affected area in the previous study An affected area of the one motion segment was calculated only by means of the length of the affected area (A) divided by the length of one motion segment (B) in the vertical direction. One motion segment was defined as the area between the upper endplate of the cephalad vertebra and the lower endplate of the caudal vertebra.

**Figure 2:**
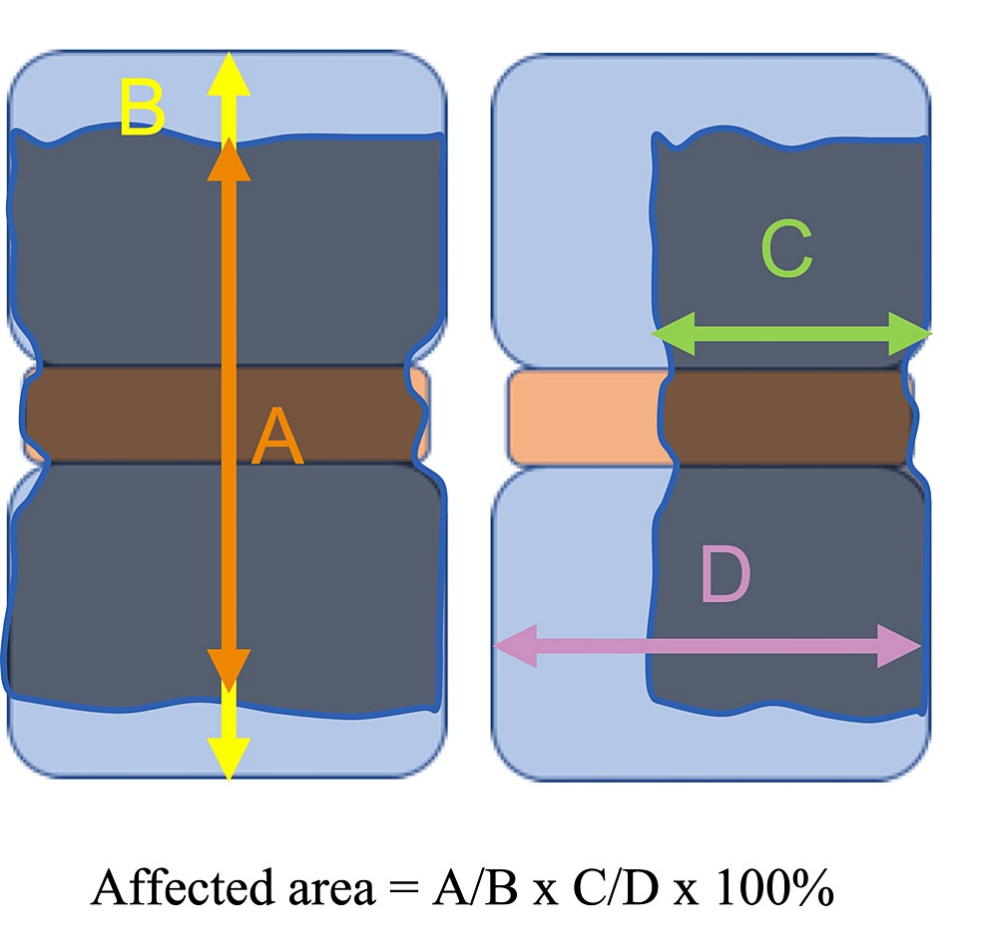
The measurement method of an affected area in the present study An affected area was measured not only in the vertical (A/B) but also in the transverse direction (multiplied by C/D). The area was rounded to the nearest 10%.

Three board-certified doctors (an orthopedist, an infectious disease specialist, and a general internist) reviewed the MRIs. The orthopedist provided a teaching video on how to review abnormal MRI findings in PVO. After following the video instructions, they independently reviewed the sagittal view of the T1-weighted image to measure the percentage involvement of the affected area in one motion segment. For validation purposes, we used the same measurement method employed in the previous study [10]. The definition of “one motion segment” was the area between the upper endplate of the cephalad vertebra and the lower endplate of the caudal vertebra, comprising two vertebral bones and one disk. The affected area showed a decreased MRI signal on T1-weighted images. The percentage involvement of the affected areas of one motion segment was measured on the MRI sagittal section, where the width of the spinal cord was maximized, that is, a cross-sectional view cut along the centerline of the spinal cord. In a previous study, measurements were conducted only in the vertical direction of the affected area (Figure 1). However, the affected area may also involve the transverse direction. Therefore, we measured the affected areas not only vertically but also transversely (Figure 2). The area was rounded off to the nearest 10% for analysis. As in the previous study, we used the same cutoff value of ≥90% of the affected area in one motion segment for validation. The diagnostic performance of the percentage involvement of the affected area was assessed in 10% increments.

**Figure 3:**
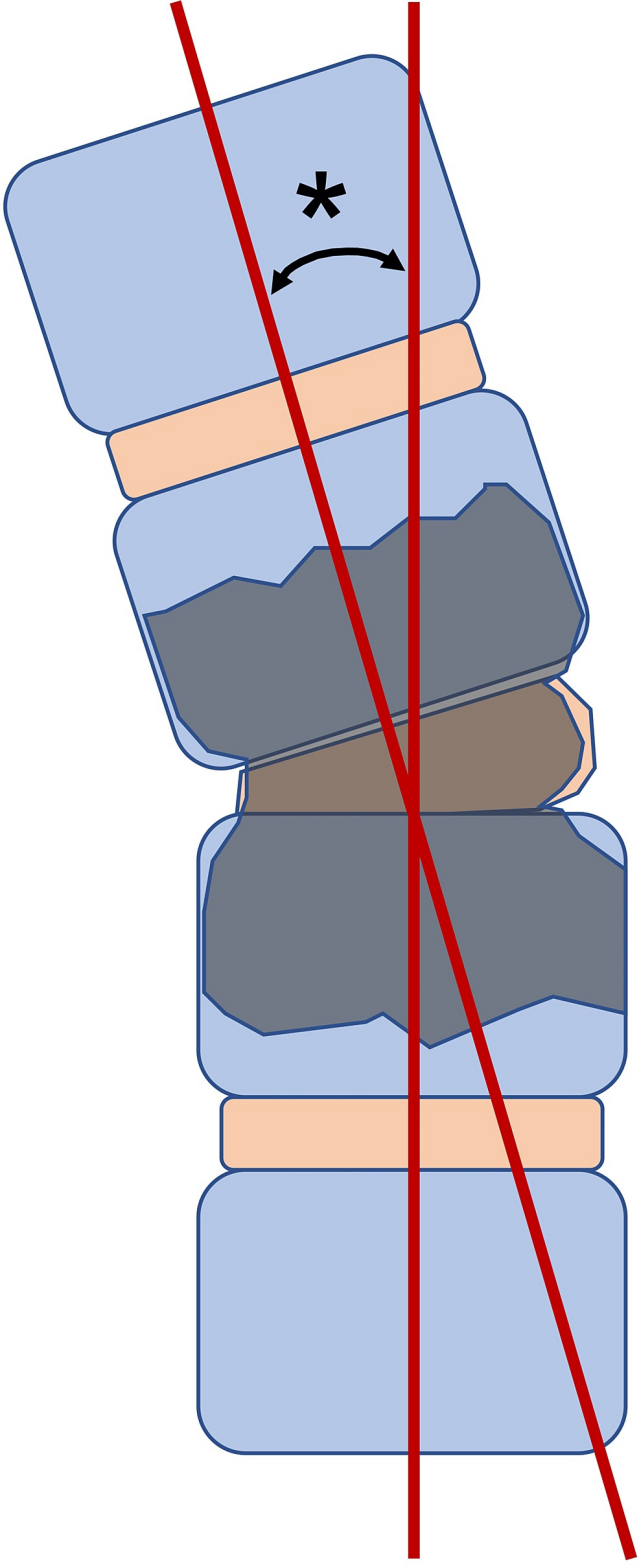
The measurement method of segmental instability * 25 degrees or more was defined as segmental instability.

As a subanalysis, we evaluated the presence of bone destruction, segmental instability, and epidural abscesses according to the Pola classification [12]. PVO is classified into three categories according to the presence or absence of neurological symptoms and imaging abnormalities. The algorithm based on the Pola classification recommends conservative or surgical therapeutic options according to the classification scheme. Segmental instability was defined as the presence of >25% change in segmental kyphosis at the infection level (Figure 3). We also evaluated osteomyelitis at an additional site.

When the interpretation differed among the three examiners after an independent evaluation of the MR images, a simultaneous review was conducted to achieve a consensus. We used the interpretations of MRIs made in this study only, apart from existing radiological reports in actual clinical practice.

Outcome

The outcome was antibiotic treatment failure, i.e., the provision of any surgical intervention that was not planned on the day of admission. It was also classified as antibiotic treatment failure when patients died despite antibiotic treatment.

Statistical analysis

All analyses were performed using a commercial statistical software (Stata, version 17 SE; StataCorp LP, College Station, TX). Based on the previous study, we expected the sensitivity and specificity of MRI findings to be 78% and 93%, respectively, and the occurrence rate of the outcome to be 40%. After setting the alpha level to 0.05, power to 0.8, and marginal error to 10%, the total sample size required for this study was calculated to range from 42 to 165 participants (42 for specificity and 165 for sensitivity) [13].

Continuous variables are expressed as mean and standard deviation (SD) and categorical variables as numbers with proportions. The predictive performance of abnormal MRI findings was evaluated with respect to sensitivity, specificity, and positive (LR+) and negative (LR-) likelihood ratios as point estimates with 95% confidence intervals (CI). The diagnostic performance of 10% increments in the affected areas was determined using a receiver operating characteristic (ROC) curve, and the area under the ROC curve was calculated.

## Results

Patient characteristics and outcome

**Figure 4:**
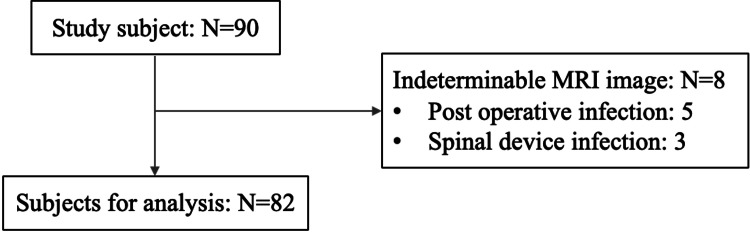
Study flow chart N, number; MRI, magnetic resonance imaging.

Among the 90 patients included in the study, 82 were eligible for analysis (Figure 4). Eight patients were excluded for postoperative infection (n = 5) and spinal device infection (n = 3). Table 1 shows the basic characteristics of the patients (mean age: 69.2 ± 9.8 years; males, 68.3%). More than one-third of the patients had diabetes mellitus: 35.4%, 34.4%, and 38.9% of all patients, patients without outcomes, and patients with outcomes, respectively. Among the 82 patients analyzed, 18 (22%) experienced antibiotic treatment failure, including one patient who died. None of the patients with antibiotic treatment failure had cerebrovascular diseases, rheumatological disorders, or malignancies.

**Table 1:** Patient baseline information and outcome SD, standard deviation; CRP, C-reactive protein, N, number.

	Total	Recovery with antibiotic treatment	Operation or death
	N = 82	N = 64	N = 18
Age: mean (SD)	69.2 (9.8)	70.3 (9.6)	65.1 (9.5)
Male	56 (68.3%)	42 (65.6%)	14 (77.8%)
Underlying comorbidities			
Diabetes mellitus	29 (35.4%)	22 (34.4%)	7 (38.9%)
Cirrhosis	3 (3.7%)	1 (1.6%)	2 (11.1%)
End-stage renal disease	13 (15.9%)	11 (17.2%)	2 (11.1%)
Hypertension	11 (13.4%)	9 (14.1%)	2 (11.1%)
Cardiovascular diseases	7 (8.5%)	6 (9.4%)	1 (5.6%)
Cerebrovascular diseases	6 (7.3%)	6 (9.4%)	0 (0%)
Rheumatologic disorders	2 (2.4%)	2 (3.1%)	0 (0%)
Malignancy	1 (1.2%)	1 (1.6%)	0 (0%)
CRP (mg/dL): mean (SD)	11.2 (9.9)	11.2 (10.4)	11.0 (8.2)
Unplanned operation	17 (20.7%)	0 (0.0%)	17 (94.4%)
Death	1 (1.2%)	0 (0.0%)	1 (5.6%)

Abnormal MRI findings and outcome

Twenty-two patients (26.8%) had a ≥90% affected area in one motion segment of the spinal bone (Table 2). Contrary to expectations, the proportion of patients with a ≥90% affected area was lower in the antibiotic treatment failure group (16.7%) than in the group without an outcome (29.7%). No patient in the antibiotic treatment failure group experienced segmental instability. The proportion of patients with bone destruction, epidural abscess, or osteomyelitis at an additional site was higher in the antibiotic treatment failure group.

**Table 2:** Abnormal magnetic resonance imaging findings MRI, magnetic resonance imaging; SD, standard deviation.

MRI findings	Total	Recovery with antibiotic treatment	Operation or death
	N = 82	N = 64	N = 18
Affected area %: average (SD)	58.9 (29.9)	58.6 (30.6)	60.0 (28.4)
Affected area ≥90%	22 (26.8%)	19 (29.7%)	3 (16.7%)
Bone destruction	15 (18.3%)	8 (12.5%)	7 (38.9%)
Segmental instability (>25°)	5 (6.1%)	5 (7.8%)	0 (0.0%)
Epidural abscess	26 (31.7%)	19 (29.7%)	7 (38.9%)
Osteomyelitis at an additional site	6 (7.3%)	3 (4.7%)	3 (16.7%)

Predictive performance of abnormal MRI findings for antibiotic treatment failure

Although the specificity of each abnormality was relatively high (70.3-95.3%), the sensitivity was low (0-38.9%), translating to poor performance in predicting antibiotic treatment failure, except for the presence of bone destruction (LR+: 3.11, 95% CI: 1.30-7.42) (Table 3).

**Table 3:** Predictive performance of magnetic resonance imaging findings for antibiotic treatment failure CI, confidence interval; LR+, positive likelihood ratio; LR-, negative likelihood ratio.

	Sensitivity (95% CI)	Specificity	LR+	LR-
Affected area ≥90%	16.7% (3.6 - 41.4)	70.3% (57.6 - 81.1)	0.56 (0.19 - 1.69)	1.19 (0.91 - 1.54)
Bone destruction	38.9% (17.3 - 64.3)	87.5% (76.8 - 94.4)	3.11 (1.30 - 7.42)	0.70 (0.48 - 1.02)
Segmental instability (>25°)	0% (0 - 18.5)	92.2% (82.7 - 97.4)	0.00	1.08 (1.01 - 1.16)
Epidural abscess	38.9% (17.3 - 64.3)	70.3% (57.6 - 81.1)	1.31 (0.66 - 2.61)	0.87 (0.58 - 1.30)
Osteomyelitis at an additional site	16.7% (3.58 - 41.4)	95.3% (86.9 - 99.0)	3.56 (0.78 - 16.1)	0.87 (0.71 - 1.08)

The presence of a ≥90% affected area had a lower LR+ (0.56) and a higher LR- (1.19) than 1.0, indicating an inverse tendency to predict antibiotic treatment failure. The same inverse tendency was observed for segmental instability. Osteomyelitis at an additional site had a relatively high LR+ of 3.56 (95% CI 0.78-16.1) but with no statistical significance.

**Figure 5:**
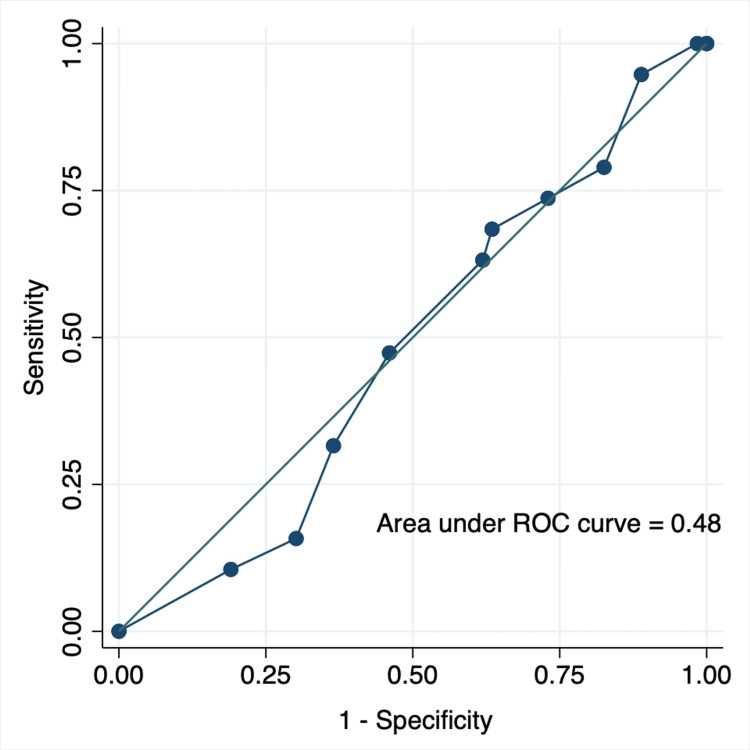
Receiver operating characteristic curve of 10% increment of the affected area ROC, receiver operating characteristic.

The ROC curve for a 10% increase in the affected area is shown in Figure 5. The area under the ROC curve was less than 0.5, indicating that the index test based on the affected area could not discriminate between patients with and without antibiotic treatment failure.

Characteristics of patients with antibiotic treatment failure

The characteristics of 18 patients with antibiotic treatment failure are presented in Table 4. One patient died of hepatic encephalopathy without surgery. Antibiotic treatment failed, and surgery was eventually required because of one or more combinations of continuous fever, worse pain, development of neurological abnormalities, high C-reactive protein level, and aggravation of imaging findings. The period between diagnosis and outcome varied: median = 31.5, interquartile range = 9.5-54.3, minimum = 1, and maximum = 68 days. Five patients underwent surgery within one week.

**Table 4:** Characteristics of 18 patients with antibiotic treatment failure M, male; F, female; PEDD, percutaneous endoscopic discectomy and drainage; CRP, C-reactive protein; MRI, magnetic resonance imaging; CT, computed tomography.

Age	Sex	Affected area ≥90%	Bone destruction	Segmental instability (>25°)	Epidural abscess	Surgical procedure	Period before surgery (days)	Reasons for antibiotic treatment failure
82	F	◯	◯	−	−	PEDD	45	Continuous fever, worse lumber pain, aggravation on MRI
68	M	◯	−	−	−	Posterior interbody fusion	18	Continuous fever, aggravation on MRI
55	M	−	◯	−	◯	PEDD	54	Continuous fever, worse lumber pain, aggravation on MRI, development of paralysis
59	M	−	◯	−	−	Laminectomy, drainage	3	Development of urination problem
46	M	−	−	−	◯	PEDD	36	Continuous fever, worse lumber pain, high CRP, aggravation on MRI
57	M	−	◯	−	−	Laminectomy, drainage	1	Development of paralysis
75	M	−	−	−	−	PEDD	65	High CRP, aggravation on MRI
70	F	−	−	−	−	Debridement, anterior interbody fusion	52	High CRP, development of paralysis
62	M	−	−	−	−	Debridement, anterior interbody fusion	13	Worse lumber pain, high CRP, development of paralysis
73	M	−	◯	−	−	Anterior interbody fusion	3	Development of paralysis
78	M	−	−	−	◯	Posterior interbody fusion	81	Development of paralysis
59	M	−	−	−	◯	Posterior interbody fusion	43	Worse lumber pain, high CRP
60	M	−	−	−	−	Anterior interbody fusion	3	High CRP, development of paralysis
70	F	−	−	−	−	Posterior interbody fusion	59	Aggravation on MRI
72	M	−	−	−	−	Posterior interbody fusion	24	Continuous fever, aggravation on MRI
54	M	−	◯	−	◯	Posterior interbody fusion	13	High CRP, positive blood culture
61	M	−	◯	−	◯	Posterior interbody fusion, laminectomy	6	Continuous fever, worse pain
71	F	−	−	−	◯	Death	57	Complicated by hepatic encephalopathy

## Discussion

Summary of findings

We evaluated the predictive accuracy of spinal MRI findings for antibiotic treatment failure, particularly to validate the accuracy of ≥90% affected area on T1-weighted MRI of one spinal motion segment, which was previously reported to be a highly accurate predictor. However, our analyses revealed that the presence of ≥90% affected area had a poor predictive performance for antibiotic treatment failure, with an inverse tendency of probability (LR+ of 0.54 and LR- of 1.19), low sensitivity (16.7%), and relatively high specificity (70.3%).

In contrast, the presence of bone destruction had a relatively high predictive performance for antibiotic treatment failure with an LR+ of 3.11 (95% CI: 1.30-7.42) and LR- of 0.70 (95% CI: 0.48-1.02), indicating that the presence of bone destruction can predict outcome occurrence [14], but its absence cannot reliably exclude outcome occurrence.

Comparison with existing knowledge

In the prior study, Hodges et al. evaluated 22 patients with PVO, including nine with antibiotic treatment failure and 13 without failure [10]. Contrary to our results of 16.7% sensitivity and 70.3% specificity, they found that ≥90% affected area of one motion segment had 78% sensitivity and 93% specificity. They concluded that early surgical intervention should be considered if patients with PVO have ≥90% involvement of one spinal motion segment. However, in our validation study, we concluded that this finding could not predict antibiotic treatment failure. This discrepancy was probably due to differences in the sample size. Type 1 errors were more likely to occur in the previous study because of the smaller sample size. Furthermore, we measured the affected area both vertically and horizontally, resulting in a more precise measurement compared to that of the previous study, which was only vertical.

Clinical implication

Since abnormal MRI findings at the time of diagnosis cannot accurately predict future antibiotic treatment failure, prediction models comprising clinical variables other than MRI findings may be required. A retrospective case series analysis of 35 patients with PVO indicated that fever and intravenous drug abuse were associated with conservative treatment failure [15]. A retrospective multicenter cohort study of 215 patients with PVO indicated that diabetes (hazard ratio (HR): 1.69), fever (HR: 1.77), osteomyelitis at an additional site (HR: 5.17), and epidural abscess (HR: 1.91) were associated with antibiotic treatment failure [16]. Osteomyelitis at an additional site and epidural abscess are MRI findings, but in our study, they did not show a significant ability to predict antibiotic treatment failure.

As significant factors for antibiotic treatment failure vary across studies, the accumulation of large-scale multicenter studies and a meta-analysis are necessary before the clinical application of the findings.

Strengths and limitations

The strength of our study is that it is the first to validate the predictive accuracy of the affected areas on spinal MRI at the time of diagnosis for future antibiotic treatment failure in patients with PVO. We used a larger sample size than that used in the previous study. In addition, we evaluated additional MRI findings and found that the presence of bone destruction was associated with a relatively high LR+ value for predicting antibiotic treatment failure.

However, this study has some limitations. First, the sample size (82 patients) was below the minimum required (n = 165) for measuring sensitivity. Data were collected from patients admitted to a single tertiary hospital over a period of 16 years. Owing to the low incidence of PVO, a multicenter study would be more appropriate to obtain an appropriate sample size. Second, we did not measure the interobserver agreement to produce a kappa coefficient. However, the instructional video created by a certified orthopedist on how to interpret abnormal MRI findings may have minimized inter-observer discrepancies. Third, the indication for surgery was not predetermined but rather depended on the judgment of each orthopedic surgeon. Therefore, the differences in indications for surgery might have contributed to the discrepancy between the results of our study and those of the previous study. A prospective study with predetermined surgical indications may be necessary for a clear outcome measurement. Finally, the affected areas in one motion segment were measured solely in two dimensions using a cross-sectional view obtained along the centerline of the spinal cord. Ideally, the affected part of the vertebral column should be calculated in terms of volume rather than area. However, as our main purpose was to validate a previous study, we had to adhere to the same measurement method to some extent. We measured the affected areas both vertically and horizontally, whereas in the previous study, the affected areas were measured only vertically.

## Conclusions

Contrary to the results of the previous study, an infection signal of ≥90% in one spinal motion segment on T1-weighted MRI at the time of diagnosis of PVO did not demonstrate sufficient predictive performance for failure of antibiotic treatment alone for PVO. High predictive performance was not observed in other MRI findings, including the size of the affected area, segmental instability, epidural abscess, and osteomyelitis at an additional site. The presence of spinal bone destruction had mild-to-moderate predictive accuracy. Accumulating studies on the predictive performance of MRI images for antibiotic treatment failure would be necessary before its practical clinical application.
